# Effects of Psychological Benefits of Greenness on Airlines’ Customer Experiential Satisfaction, Service Fairness, Alternative Attractiveness, and Switching Intention

**DOI:** 10.3389/fpsyg.2022.834351

**Published:** 2022-04-27

**Authors:** Suk Ha Grace Chan, Xiaocheng Vicky Zhang, Yifan Betty Wang, Zhaofeng Mason Li

**Affiliations:** Faculty of International Tourism and Management, City University of Macau, Macau, China

**Keywords:** alternative attractiveness, experiential satisfaction, green airlines, service fairness, switching intention

## Abstract

In the context of climate change, this study uncovers the role of green airlines’ social responsibility in conjunction with the consumers’ switching behavior while considering the effects of latent variables, including green psychology, airline corporate image, green experimental behavior, green service fairness, green alternative attractiveness and switching intention, were examined in the study. In a highly competitive service environment, an organization needs to understand how passengers perceive its corporate image, satisfaction, fairness attractiveness, and behavior of switching intention. The predicted relationship was based on partial least squares structural equation modeling of a convenience sample of 615 valid datasets collected from individuals who used green airline services in China. The findings show that the psychological benefit of greenness, only warm glow, is the main driver of airline corporate image. Furthermore, airline corporate image, green service fairness, and green alternative attractiveness support passengers’ green experiential satisfaction. The evidence demonstrates that green experiential satisfaction and green alternative attractiveness have significantly positive effects on switching intention. However, green service fairness has no significant effect on green switching intention. This study contributes to the literature by understanding airline customers’ perception of the complex relationship in the green constructs. This finding can help marketers facilitate and develop their external communication and craft their image to retain their existing or potential customers.

## Introduction

Over the last two decades, global warming and environmental pollution have created a high awareness in the hospitality industry. The attention for environmental protection continues to rise (Trenberth et al., 2014; Singh and Sharma, 2017). The hospitality sector plays a role in social responsibility and commits to protecting the environment as one of its marketing strategies. The aviation industry is the basic and leading industry of economic development and an important carrier for promoting the development of the tourism economy. Hospitality products are wrapped in a green image to provide psychological benefits, hoping to provide the customers with satisfactory values and needs (Wu and Cheng, 2018a).

As more environmental regulations have been implemented and individual environmental awareness has increased, an increasing number of travelers have been searching and buying green tourism products (Han et al., 2011a; Han and Hwang, 2017). Many customers are willing to buy more environmentally friendly products or services provided by the hospitality sector (Liu et al., 2017). Green airline products have gained popularity in the tourism market, and green airlines’ sustainable practices may affect the competitiveness in the market (Khare, 2014). Rajiani and Kot (2018) stated that new practices in familiarizing green air traveling deliver comfort for career-oriented clusters. These clusters are presented as active, calculating, and rational consumers who carefully allocate scarce resource clusters until their cost and benefit analysis is confirmed satisfactory.

Airlines need to create a stable performance. They are not limited to seeking new opportunities, they also need to create highly innovative products and services (Kong and Ibrahim, 2018). Previous scholars identify organization with capacity to innovate will be able to speed up environmental challenges and better than those non-innovated organization (Miles and Snow, 1978; Cingöz and Akdogan, 2013). Organization gain innovative service, it may enhance the service processes and increasing competitiveness among the industry. As a result, they provide effective service quality and benefits to their customers and come up with a strong relationship and customer retention. They avoid switching to other competitors (Kong and Ibrahim, 2018). Many airlines provide innovative services by adopting a green image and the psychological benefits from the green products indicate trust and reliability to the potential buyers. Product satisfaction may lead to buying decisions (Tran, 2020). Although the issues seem important, research on the psychological benefits of greenness, which affect airline travelers’ switching behavior, is limited. The extent of customer experiential satisfaction, service fairness, and green alternative attractiveness that may have the potential to lead to switching intention is also under discussion. Previous scholars have highlighted that green images are associated with experiential satisfaction. They have demonstrated that green experiential satisfaction plays a key role in influencing satisfaction (Wu et al., 2016, 2018b; Wu and Cheng, 2019) and identified that green experiential satisfaction may influence green switching behavior in the tourism products. Nikhashemi et al. (2017) identified that switching intentions are predictors of switching behavior, indicating that switching intentions positively influence satisfaction and switching behavior. However, limited studies have paid attention to green service fairness and green alternative attractiveness, which may potentially affect green experiential satisfaction and switching intention. The current academic research on the measures of carbon emission reduction in airlines has mainly focused on technology (Lou et al., 2015) and policy (Hwang and Choi, 2018). Other studies have focused only on the green perception of the choice of the airlines (Hagmann et al., 2015). Carr (2007) argued that in relational service contexts, customers’ perception of service fairness is vital for satisfaction. Service fairness may lead to satisfaction and service encounters (Olsen and Johnson, 2003; Zhu and Chen, 2012). Nysveen et al. (2018) highlighted that service fairness has a relationship with switching behavior. However, in the airline sector, the importance of green alternative attractiveness, which may potentially affect green experiential satisfaction and switching intention, has not been mentioned in the studies. In the context of green airlines business, knowledge of the relationship between green airline switching intention and psychological benefits of greenness with experiential satisfaction, green service fairness, and green alternative attractiveness, are limited. Vuong et al. (2022) highlighted that knowledge management from social sciences plays a pivotal role in positively changing human behavior and suggested evidence-based policymaking in communication. It can attain more insights into perceptions and positive buying decisions (Chan et al., 2021).

Airline practitioners should understand the consequences of switching behavior in green airline services to provide all the benefits and service satisfaction. Therefore, this study aims to fill the aforementioned research gaps and is expected to achieve the following objectives:

• To examine the relationship between the psychological benefit of greenness and airline corporate image.
• To explore the relationship between airline corporate image to green experiential satisfaction and switching behavior.
• To investigate the constructs of dimensions of green experiential satisfaction, green service fairness, green alternative attractiveness, and airline switching intention perceived by airline customers.

The study contributes by extending the green theory from theoretical and practical perspectives to the airline sector. From the theoretical approach, the study proposes a unique construct, which includes psychological benefits affecting airlines’ green image, airlines’ green image affecting green experiential satisfaction and airline switching behavior. The dimension of the constructs also examines the green experiential satisfaction, green service fairness, green alternative attractiveness, and airline switching behavior, incorporated in a relevant marketing model. The study’s unique contribution is to gain an understanding of the airline customers’ perception of the complex relationship in green constructs. From the practical perspective, industry practitioners may understand how customers perceive airlines’ green benefits, corporate image, green experiential satisfaction, service fairness, alternative attractiveness, and switching intention. The study can help the airlines to stimulate green switching behavior through the five determinants mentioned (i.e., psychological benefits of greenness, green corporate image, green experiential satisfaction, green service fairness, and green alternative attractiveness). This finding can help marketers facilitate and develop their external communication and craft their image to retain their existing or potential customers.

## Literature Review

### Green Airline Marketing

Green airline marketing has emerged with the growing environmental awareness across all levels of society and with the rise in the segment of green consumers (Kumar, 2016). The role of market communication concerning the eco-positioning of brands or corporations is important in the sustainable marketing mix (Khoo and Teoh, 2014; Migdadi, 2020). An organization can influence its green brand positioning by actively communicating with the environmental attributes of the brand in comparison with the competitors’ brands (Hartmann et al., 2005). Khoo and Teoh (2014) noted that most of the aircraft emission level is affected by the aircraft load factor, fuel efficiency, cabin density configuration, aircraft size, and service frequency. Based on the effectiveness of the airlines’ green operation strategies, Migdadi (2020) developed an effective categorized pattern with low, low-to-moderate, and high emphasizer patterns through fuel-saving actions (i.e., flight route management and flight weight management), energy-saving actions (i.e., upgrading and replacing of facilities, vehicle and energy design, and transportation management), waste management and recycling actions (i.e., recycling, upcycling, and reusing waste), and water management actions.

In addition, utilizing advertising and the whole communication mix to address environmental credibility and concern can also be regarded as useful tools in creating positive eco-positioning among air travelers. Peattie (2010) stated that individual psychographic concerns of environmental protection would translate into changed consumption behavior, which is relatively consistent across different consumption spheres. However, as consumers often consider the premium price for an environmentally superior product, marketing green products and services requires different strategies than the traditional ones (Dangelico and Vocalelli, 2017). Although green products are crucial to the environment, green airlines’ psychological benefits to customers are continuously being ignored. The psychological benefits of green content create an advantage for consumers. On the other hand, this content can let customers participate in protecting the environment (Han et al., 2019; Trang et al., 2019). Therefore, further examining and having a better understanding of how customers perceive green benefits are worthwhile for organizations to meet the satisfaction of the targeted green customers.

### Airline Corporate Image

The overall brand image indicates the global and general beliefs and perceptions that patrons develop based on diverse sources from the acquired and processed information on a particular brand (Assael, 1984; Han et al., 2019).

Green brand image has become a popular topic in society. To obtain this image, a brand should be able to differentiate itself from other brands through consistent and dedicated activities designed for green actions (Lin and Zhou, 2020). An organization can provide five desired benefits to develop green marketing, namely complying with environmental pressures, raising corporation competitiveness, enhancing the corporate image of a company, seeking a new market for opportunities, and enhancing product value (Chen, 2010; Singh and Pandey, 2012). Consumers perceive a green brand image as a correlation between a brand with environmental commitments and environmental concerns (Chen, 2010). Hinnen et al. (2017) stated that consumers’ willingness to pay a premium price for greenness contributes to the corporations in air travel when cost-effectiveness exists compared with purchasing a regular product. A previous study also indicated that the green market segment should focus on strong competitive advantages for products and services, including quality and prices (Borin et al., 2013). Therefore, understanding the customers’ perception of green airlines may provide an advantage for airline practitioners in promoting their services.

#### Green Service Innovation and Customer Satisfaction

The competition in the Airlines industry, without service innovation, is a serious threat for the industry. The growing customer acquisition costs and increased customer expectations need the airlines to create value in the way of adopting innovative services as a response to the increasing competitive pressure and developing service innovation to ensure customer satisfaction and retention (Kong and Ibrahim, 2018). Service innovation is defined as the process of developing and releasing a new or important product or service to meet customer needs and wants (Al-Otaibi and Al-Zahrani, 2009). The green concept adds value to the environment, enlarges the green tourism products, and establishes a new management system (Zainal Abidin et al., 2011). The airlines adopted a green management system and changed their business practices and increased external communication (Rajapathirana and Hui, 2018). The change in service innovation may affect the technical and social systems of an organization. The change might affect their performance and help the customers better understand the types of capabilities that can result in competitive advantages.

The rationale of service itself comes up with “product” and “process” for service and manufacturing (Tether et al., 2002). When design a service innovation for tourism organization as multidisciplinary process of designing, realizing, and marketing combination of existing new service and products. The major task is creating value for customer experience and providing benefit to the service organization.

Very often, customers evaluate the service product provided by the tourism organization after they purchase the product (Choi and Kim, 2013). Davis and Heineke (1998) highlighted the conceptual idea of the “confirmation/disconfirmation” theory of customer satisfaction. The customers’ level of satisfaction and their perception of tourism organization performance may have a relationship in their future buying decision. Create big gaps found between their perception and actual performance, it may lead to dissatisfaction and repetitive of the service or even lead to switching behavior (Palawatta, 2015). According to Hilal (2015), identifying the introduction of new innovative services may improve the service productivity and need to ensure the innovative product and service that their appropriately price to attract and provide satisfaction to customer. Other studies found customer satisfaction in relation to service innovation and customer value (Weng et al., 2012). Therefore, green airlines’ psychological benefits in innovation product cannot be ignored.

### Modeling Green Customer Behavior

Bhattacharya and Sen (2004) categorized green marketing theories and group consumer-level theories into six categories, namely value and knowledge, beliefs, attitudes, intentions, motivations, and social confirmation. Among the existing models, the theory of reasoned action (TRA) and the related theory of planned behavior (TPB) are most commonly applied to green consumerism (Peattie, 2010). TPB is the extension theory of TRA. The fundamental tenet of the TPB is that individuals tend to make reasoned choices and choose alternatives that have the highest benefits and least costs or negative effects to themselves (Li and Wu, 2019). Ajzen (1991) stated that behaviors are shaped by intentions, which, in turn, are driven by consumers’ attitudes, subjective norms, and perceived behavioral control. Chen (2016) also stated that TPB is successful in forecasting and interpreting individuals’ intentions and behavior in a wide range of environmental causes. However, some scholars have argued that TRA and TPB still have limitations in several aspects. A large intention with limited reaction highlights the contrast of traditional TPB, which may ignore other essential factors, such as unconscious motivation, spontaneous choices, and external temptation. Therefore, researchers have attempted to modify the model through extended variables or models and enhance its strength in explaining TPB (Chen, 2016; Li and Wu, 2019).

In addition, based on the green marketing category of consumers’ intention, the rational choice theory, consumer choice theory, and acquisition–transaction utility theory explicitly focus on economic intentions (Bhattacharya and Sen, 2004). Intentions are present as the predominant individual desires and are initially formed in thoughts before they can be achieved. Notably, however, green airline marketing mainly investigates positive economic intentions, such as revisit, purchase, and repurchase intentions after satisfying the consumers’ needs. However, some scholars have argued that the judgment of consumer satisfaction should be divided into positive and negative emotions. Moreover, they are not only opposite in concept but also two extremes in an independent space. Martins et al. (2013) presented that satisfaction has an inverse influence on the switching intention, indicating that satisfied consumers are less likely to switch than unhappy ones. Satisfaction is considered a function of the perceived performance relative to the consumers’ prior expectations (Chiesa et al., 2020).

### Psychological Benefits for Green Users

Many scholars have identified using green services that provide green benefits and a more environmentally friendly approach (Wu et al., 2016; Xie et al., 2019). The previous scholars have defined the concept of psychological benefits using environment services, indicating spiritual benefit and comfort for customers using airline services (Gwinner et al., 1998; Hwang et al., 2019). Hartmann and Apaolaza-Ibáñez (2009) highlighted the psychological benefits, including warm glow, self-expressive benefits, and nature experience. When people think that they are concerned for the environment, they might create awareness of warm glow and take social responsibility. Spielmann (2020) recently posited that warm glow is the customers’ thinking that they will be rewarded for their environmentally friendly behavior, taken as intrinsic satisfaction.

Self-expressive can be defined as customers’ benefit to signal concerns about environmental problems. Customers hope to express themselves to protect the environment and more likely want to travel in a green airline, giving them a high level of satisfaction and self-expressive benefit (Hartmann and Ibanez, 2006; Hu, 2012).

Nature experience is a vital element in psychological benefits. Understanding the nature experience and the enhancement of well-being perception are important. Thus, people spend time in a natural setting, and they can recover from stress during their short stay in the natural environment (Hwang et al., 2019). Customers have a high awareness of nature experience, and they will more likely prefer green services or choose green airlines (Hartmann and Apaolaza-Ibáñez, 2012).

Therefore, the above three elements provide support to psychological benefits to green users. Given a large number of benefits, many customers may not want to switch their environmental-friendly products (Wu et al., 2018b). To maintain the competitiveness of the service environment, airlines need to utilize green branding and decrease customer switching behavior for environmental concerns (Chen, 2010) and retain their loyalty to green customers.

### Green Switching Intention and Satisfaction

In the service context, previous scholars have identified trust and satisfaction as constituting relationship quality (Farooqi, 2014). The former is more likely in an eco-friendly organization, whereas the latter is with the suppliers. For example, hospitality customers will evaluate the environmental-friendly context based on their experience and satisfaction. Jung and Yoon (2012) proposed that the variation needs have moderated the effect of satisfaction on switching intentions. Conversely, Setiyaningrum (2006) claimed that the variation needs do not moderate the effect of satisfaction on switching intentions when applied to different services.

Customer satisfaction is crucial in marketing theory. A service organization needs to provide to customers’ needs and desires. Customers make judgments in terms of service features and their attributes (Back and Parks, 2003). If the performance exceeds customers’ expectations, they will be satisfied, otherwise, they will be displeased with the services or switch to another service provider. Han et al. (2011b) mentioned that a customer-perceived service organization’s lack of attractive alternatives is an important constraint on the customers’ switching intention. Service switching will harm customer loyalty, retention, and repurchase intentions. Customer loyalty indicates a customer’s mindset of customer value and company resources and skills. Organizations can provide high service quality skills and motivate consumers to strengthen their relationship with their service provider (Hess et al., 2003; Bell et al., 2005). Anton et al.’s (2005, p. 139) proposal that an organization’s green commitment can be understood as the desire to develop and maintain long-term exchange relationships—a desire that materializes in the realization of implicit and explicit promises as well as their sacrifices, and the economic and social well-being of the parties having some interest in the relationship. An airline’s green commitments can gain the interest of the customers and offer frequent communication and information. Consumers can obtain more information and foster their loyalty, and as a result, they will not switch their loyalties. Some cases indicate that organizations keep customer loyalty so they do not contemplate other competitors (Wathne et al., 2001).

Furthermore, the high quality of the products can motivate customer loyalty, but price fairness is the reason which leads to switching intentions. According to Keaveney (1995), customers switch because they are dissatisfied with the price they paid. They may feel the price is unfair or they might have other options of fair pricing. Therefore, the price-related issue is one of the issues which leads to switching behavior.

Therefore, the present study provides a great understanding of the psychological green benefits linked to an airline’s corporate image and its green experiential satisfaction, green service fairness, green alternative attractiveness, and switching intention.

### Research Model and Hypothesis Development

**FIGURE 1:**
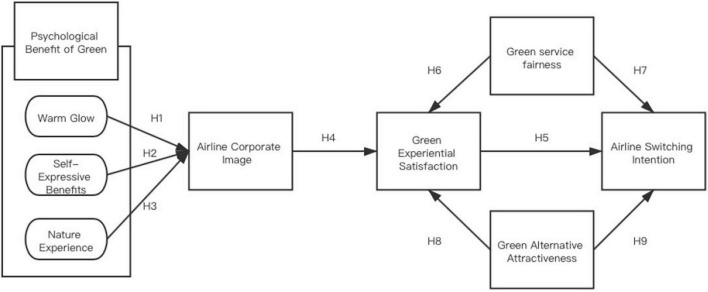
Conceptual research model.

Based on the above discussion, Study proposes a conceptual framework for this study (Figure 1). We use a multidimensional model indicating an airline providing psychological benefits of greenness, airline corporate image, green experiential satisfaction, green service fairness, green alternative attractiveness, and airline switching intention.

#### Psychological Benefit of Greenness

Psychological benefit is regarded as the post-purchase behavior, which is defined as an individual’s spiritual comfort generated after buying a brand’s products and services (Hwang et al., 2019). Vuong (2021) highlighted that environmental value will reshape human behavior in the business sector. The previous studies have provided three dimensions to measure the psychological benefits for green brands, namely warm glow, self-experience, and nature experience (Hwang and Choi, 2017; Lin et al., 2017b; Hwang et al., 2019; Liao et al., 2019).

#### Warm Glow

The warm glow of giving posits that impure altruism can motivate individuals to contribute to the public good through pro-environmental behavior, which is supported by the pro-social behavior theory (Aaker, 1999; Hwang et al., 2019). This concept has received increasing interest in the green brand domain (Lin et al., 2017b). Hwang and Choi (2017) confirmed that warm glow has a positive influence on the overall brand image. Similar research has also indicated the importance of warm glow factors, which affect consumers’ psychological attitudes positively toward the use of brands (Hartmann and Apaolaza-Ibáñez, 2012; Liao et al., 2019; Boobalan et al., 2021). Based on the above discussion, we propose the following:

H1: Warm glow has a positive influence on the green airline corporate image.

#### Self-Expressive Benefit

The self-expressive benefit concept is based on the signaling theory, which states that individuals discover psychological benefits through self-expressiveness. Correspondingly, they tend to indirectly express the preferred information to others (Ahmad and Thyagaraj, 2015; Boobalan et al., 2021).

A brand’s benefits refer to the consumers’ perceptions of a brand based on what they can attain for the product attributes (Lin et al., 2017a). Functional brand benefits are usually correlated with consumers’ functional needs to easily develop positive brand attitude for consumers (Lin et al., 2017a). For instance, customers are comforted by the tendency to jointly protect the environment for sustainable development and convey positive information. In this case, individuals are more likely to have a high level of self-expressive benefits and provide a positive attitude toward high-signaling products or services labeled in “green,” “eco,” or “sustainability” (Lin et al., 2017a; Hwang et al., 2019).

Aaker (1999) noted that as individuals act differently in varying situations, the empirical support of self-expressive research should be based on context. Studies in different areas have reported conflicting findings. For instance, Hartmann and Apaolaza-Ibáñez (2012) could not find the linkage between self-expressive benefits and general brand attitude in the context of brands linked to the supply of electricity. However, fields such as emotional nature experience (Hartmann and Apaolaza-Ibáñez, 2008), charity (Andreoni, 1989), and energy-saving appliances (Liao et al., 2019) have a positive linkage. This study examines self-expressive benefits and green brand image under a green airline scenario.

H2: Self-expressive benefits have a positive influence on green airline corporate image.

#### Nature Experience

Hwang et al. (2019) posited that nature experience serves as the most important psychological benefit in an eco-friendly topic. Nature experience has been investigated in diverse fields, such as the dimension of experience and tourists’ purchase intentions (Jamrozy and Lawonk, 2017), experience and worth of money experience, and satisfaction (Gallarza and Saura, 2006; Williams and Soutar, 2009; Wu et al., 2014; Sharma and Nayak, 2019). Moreover, the satisfaction of a brand image in an eco-airline domain was highly limited in the previous studies. However, individuals perceiving a high level of nature experience tend to have a positive thinking toward the corporate brand image (Hwang and Choi, 2017). Thus, this study proposes the following hypothesis:

H3: Nature experience has a positive influence on the green airline corporate image.

#### Airline Corporate Image and Green Experiential Satisfaction

Wu et al. (2018a) stated that the concept of green experiential satisfaction is the novel concept that evaluates consumers’ overall experience satisfaction based on their experience places. Nysveen et al. (2018) found strong empirical support for the relevance of perceived green image and experience (tested through sensory experience, affective experience, cognitive experience, relational experience, and behavioral domains) in the hotel sector. Gelderman et al. (2021) found that the green corporate image has a significant effect on consumer satisfaction in the business-to-business context. Furthermore, green product quality, green product price, and salespersons’ green expertise have shown positive effects on green experiential satisfaction. Han et al. (2018) confirmed that experiential satisfaction on airport duty-free shopping has a positive influence on consumer loyalty. In addition, the satisfaction with airport duty-free shopping has significant associations with purchase desire in duty-free shops.

The previous studies have found that green brand image produces some valuable outcomes, such as green trust, satisfaction and brand equity, word-of-mouth intention, and green competitive advantage (Lin and Zhou, 2020). Lin and Zhou (2020) noted that the utilitarian environmental benefit and green brand innovations have a direct effect on green brand image. Psychological benefits (e.g., warm glow, self-expressive benefits, and nature experience) ensure the perceptual effects in green brand positioning (Hwang and Choi, 2017; Lin et al., 2017a; Hwang et al., 2019). Generally, functional benefits’ cognitive and affective brand attributes affect consumers’ judgment of the overall image. Thus, green brand image can affect consumer satisfaction and loyalty (Hartmann and Apaolaza-Ibáñez, 2008; Lin et al., 2017a).

H4: Green airline corporate image has a positive influence on green experiential satisfaction.

#### Green Airline Experiential Satisfaction and Switching Intention

Park et al. (2015) mentioned that experiential satisfaction can be measured by consumers’ favorable and unfavorable factors. Favorable factors, such as word-of-mouth communication, purchase intention, and price sensitivity, are conducted in various academic fields and have been investigated by many researchers (Chang and Fong, 2010; Park et al., 2015). In comparison, unfavorable factors, such as complaints and switching intentions, have been investigated by few researchers (Wu and Cheng, 2018b).

Switching intention is defined as the possibility of transferring consumers’ existing transactions with an organization to a competitor (Liang et al., 2018). Wu and Cheng (2018b) found that satisfaction has a direct influence on the switching intention in tourism destinations. Tran (2020) highlighted that the consumers’ perceived risk toward an organization reduces consumers’ perceived satisfaction. The decrease in consumers’ perceived risks toward an organization may increase their purchase intention. Therefore, consumer perceived satisfaction was the most crucial factors that lead to purchase intention. Liang et al. (2018) considered satisfaction in the AirBnB concept and confirmed that transaction- and experience-based satisfaction directly influence switching intention. Based on these studies, if customers are satisfied with their experience, then their intention to switch will be lesser. Therefore, the present study proposes the following hypothesis:

H5: Green experiential satisfaction has a negative influence on airline switching intention.

#### Green Service Fairness, Green Experiential Satisfaction, and Switching Intention

The concepts of service fairness and equity, which originated from the equity theory and are extensively used, can be used synonymously (Cappelli and Sherer, 1988; Wu and Cheng, 2018b). Consumers should equally divide products and services in terms of their satisfaction and what they deserve (Wu and Cheng, 2018a). Through consumers’ previous flight experience or other replacement transportations, service fairness is a comprehensive judgment based on their fairness standards (Seiders and Berry, 1998). Sarker et al. (2019) concluded that the previous literature regarded consumer-based brand equity (CBBE) and conceptualized consumer-based service brand equity (CBSBE) models, which are suited for the Airlines industry. They claimed that the concept of service brand equity for consumer flight experience is a composite mission. This mission makes practitioners pay attention to consumers’ understanding, feelings, and brand stimuli, such as in-flight services, employee interactions, and price, which have not been presented in the CBBE model. Therefore, they composed a more comprehensive model to examine airline service equity, that is, stimuli (e.g., airline service and direct service experiences, and brand consistency) and organism (brand awareness, brand meaning, and perceived value).

Recent studies have found that service fairness plays an essential role in satisfaction and switching intention aspects. Wu et al. (2016) summarized the previous literature and concluded that research switching intention, experiential satisfaction, and equity have been investigated in the restaurant, hotel, golf, chain restaurant, tourism, and tourist destination fields. Setiawan et al. (2020) stated that price fairness is based on consumers’ expectations on the price that suit equivalent service quality and are even fairer than those offered by other airlines. Jiang and Zografos (2021) regarded that green service fairness constitutes an important criterion for allocating scarce resources among self-interested practitioners. Many consumers will evaluate green products to determine whether the true value of the green offerings is justifiable concerning their inputs to acquire the service. Therefore, investigation of fairness perception will provide a new theoretical direction to confirm consumers’ behavioral response to green service offerings and their satisfaction in purchasing green products (Yuen et al., 2018). To the best of our knowledge, the linkage has not been investigated in the sustainable airline field. Therefore, we propose the following:

H6: Green service fairness has a positive influence on green experiential satisfaction.

H7: Green service fairness has a negative influence on airline switching intention.

#### Green Alternative Attractiveness, Experiential Satisfaction, and Green Airline Switching Intention

When consumers balance the alternatives of price, value, service, quality, or other essential elements and conclude that other airlines have better performance, green alternative attractiveness is formed (Han, 2015). However, when superior competition is lacking, consumers might not have any other choice: to stay or leave (Han, 2015). In other words, when consumers in this area, who are knowledgeable, emphasize green (higher demand rate) and green airlines are in an oligopoly monopolized market (few viable alternatives), the switching intention may decline.

Ortegon-Cortazar (2019) offered a list of alternative attractiveness factors based on a multidimensional analysis of eco-natural resources in malls, including, but not limited to, access to the malls, the variety of offerings, clients, the physical design of the malls, luxurious feeling, and eco-natural environment. Customers are regularly attracted to strong alternatives, particularly when they perceive the relative merit of competing with the alternative’s price, value, location, service, or quality. According to Sharma and Peterson (2000), customers are likely to terminate an existing relationship with a service provider and go to a new provider when they perceive that the alternative is more attractive. Thus, customers switching to the other service providers is expected in exchange for positive service, price, and image (Kim et al., 2004). Nagengast et al. (2014) confirmed that alternatives lacking attractiveness will lead to switching covered moderating nature, especially for the relationship between repurchase intention. Indeed, the result from switching to a potentially more satisfying alternative might be weakened by enhancing the switching costs, and increasing individuals’ perceived level of switching costs (e.g., reducing alternatives’ attractiveness) and are thus likely to undermine the satisfaction repurchase intention link. Han (2015) identified that the relationships between guests’ pro-environmental intention for green hotels and their direct predictors are under the influence of their perceived level of the attractiveness of non-green alternatives. The result confirmed that customers’ perceived non-green alternatives are less attractive than green lodging products. Han (2015) utilized the extended TPB model to investigate the moderating effect of non-green alternative attractiveness. The author found that the alternative is less attractive when consumers consider their attitude, perceived behavioral control, and moral obligation. Wu and Cheng (2018b) noted that because of green convention attendees, green alternative attractiveness has a significant effect on green switching intention. However, the relationship between green alternative attractiveness and green experiential satisfaction is not empirically supported by consumers. This relationship cannot represent green airlines. Therefore, we propose the following:

H8: Green alternative attractiveness has a positive influence on green experiential satisfaction.

H9: Green alternative attractiveness has a positive influence on airline switching intention.

## Research Methodology

The study systematically provided an overview of the previous literature to determine the proper items suitable for research problems and research objects. The participants were informed clearly about the research objectives and expected outcomes. If a participant did not complete the survey, then the data was not used. Kovaova and Lewis (2021) mentioned that any survey that did not reach greater than 50% of completion should be removed from the subsequent analysis to ensure quality. Quantitative research was used as an appropriate method in analyzing the conceptual model to examine our hypothesis, and the rationality of the hypothesis was verified through data collection and analysis. The descriptive statistics of the questionnaire items were measured by a seven-point Likert scale, “1” indicating “strongly disagree” and “7” indicating “strongly agree.”

Each construct was measured using multiple measurement items (Churchill, 1979). The previous study verified that three items or more to represent each construct present a more reliable result (Baker and Crompton, 2000). In this study, the measurement of the psychological benefit of greenness (with warm glow, self-expressive benefits, and nature experience dimensions) was based on Hwang and Choi (2017), Lin et al. (2017a). The seven questions to assess the green corporate image were derived from Hwang and Choi (2017), Wu et al. (2017), Boobalan et al. (2021). The assessment of green experiential satisfaction, and green alternativeness and green service fairness was initially from Wang et al. (2018), Wu and Cheng (2018b), respectively.

To avoid the difficulties caused by improper design in the formal survey, a preliminary test of the questionnaire should be conducted before the formal survey. Through the preliminary modification of the questionnaire, the accuracy of the study was improved, and the questionnaire could be distributed to the target population. We could also assess the accuracy and inertia of the possible responses. As this study is based on the people who have taken green aviation and have the basic knowledge of green aviation, the survey scope is extremely wide.

In the preliminary test, the researchers sent questionnaires to industry practitioners with green knowledge using an online platform. The main reasons for selecting them as the prediction object are as follows: First, industry practitioners have a basic concept of what green airlines are, and they can provide some advice for improving the questionnaire to minimize the bias, which can enhance the effectiveness of the questionnaire. The original questions were in English. Therefore, a bilingual expert was invited to check the translated questionnaire to ensure its validity. Back translation was adopted to increase credibility.

The study was initially designed with a 37-item questionnaire. The ratio of the item to the number of pre-testers was approximately 1:5, which is most suitable to ensure the recovery rate (Wu et al., 2014). According to this ratio, 200 copies were finally collected, and 185 valid questionnaires were collected with an effective recovery rate of 92.5% to ensure research quality (Kovaova and Lewis, 2021).

Factor analysis is needed before determining the questionnaire, which helps determine whether the dimensions can be empirically verified. Generally, exploratory factor analysis is used for verification. Through the exploratory factor analysis of 37 items, four items were eliminated (i.e., “Overall, I am happy with this eco-friendly airline because it is environmentally friendly;” “I feel like a superior consumer when I choose an eco-friendly airline;” “With an eco-friendly airline, people around me can observe that I am aware of ecological development;” and “I have already changed eco-airlines several times.”).

As a result, 33 items were retained. The convenience sampling method and online survey were used as a tool to collect the target participants. For the received data, partial least squares structural equation modeling (PLS-SEM) through SmartPLS 3.3.3 software was used to conduct confirmatory factor analysis (CFA) and hypothesis testing.

## Results and Discussion

The selected participants belong to China and are over 18 years who have been choosing green flights. To be sure of the reliability and accuracy of the result, the participants went through a rigorous verification process with two filtering questions to ensure that they could answer the questions (Kovaova and Lewis, 2021). The online questionnaire was uploaded to the Wenjuanxing platform *via* a WeChat group (one of the popular social media platforms in China) and was sent to the participants. Two filtering questions were asked to see whether they had knowledge about green flights and had traveled with green flights before. If they answered yes on both questions, they could continue with the questionnaire; otherwise, the survey was to end. The parameters of the study were clearly described before they started answering the questionnaire, and a pilot test could ensure the clarity of the survey. The participants were required to fill up the questionnaire that had listed a series of questions that influence green airline experience based on passengers’ psychological behavior, brand, experiential satisfaction, airline alternatives, service fairness, and airline switching intention.

Data collection was completed within 3 months, from April to July 2021. Given that many cities were in lockdown due to the Corona Virus Disease (COVID-19) pandemic, we adopted an online questionnaire to ensure a safe environment and minimize the spread. We provided the questionnaire link and sent it to the respondents, and we adopted convenience sampling for data collection.

A total of 684 questionnaires was received in this study, of which 615 were valid after incomplete information was removed. The response rate was 89.91%. Li (2016) recommended at least 100 samples for data analysis. When the sample size was greater than 200, the analysis results were better. When determining the sample size according to the observed variables, the ratio of the observed variables to the sample size should be between 1:10 and 1:15. A total of 33 variables were observed in this study. The acceptable sample size should be above 330. The samples collected in this study were larger than the minimum sample size of 330.

In the data statistics of the respondents, the males accounted for 46.18%, and females accounted for 53.82%. Most of the respondents were aged between 18 and 29, accounting for 54.31%, with a diploma or bachelor’s degree (74.8%). In addition, the interviewees were mainly students (39.51%), and took green flights one to two times, accounting for 57.72%. Table 1 shows the details of the demographics of the respondents.

**TABLE 1:** Survey respondents’ demographic profiles (*n* = 615).

Items	Statistics	Frequency	Percentage
Gender	Male	284	46.18
	Female	331	53.82
Age	18–29	334	54.31
	30–39	72	11.71
	40–49	76	12.36
	50–59	77	12.52
	60 or above	56	9.11
Education	High school and below	66	10.73
	Diploma or bachelor’s degree	460	74.80
	Master’s degree	72	11.71
	Doctor’s degree and above	17	2.76
Occupation	Official	30	4.88
	Education	28	4.55
	Students	243	39.51
	Sale/marketing	44	7.15
	Services	44	7.15
	Business owner	32	5.20
	Self-employed	75	12.20
	Internet industry	33	5.37
	Retiree	36	5.85
	Others	50	8.13
Monthly personal income (RMB)	3000 and below	238	38.70
	3001–6000	135	21.95
	6001–9000	100	16.26
	9001–12000	73	11.87
	12001–15000	40	6.50
	More than 15000	29	4.72
Frequency of choosing green airlines	1–2 times	355	57.72
	3–4 times	169	27.48
	4 times or more	91	14.80

*RMB 6.54 = USD 1.00 (at the time of writing).*

### Data Result

#### Confirmatory Factor Analysis

In this study, to confirm the reliability and validity of the scales, we tested the measurement model fit by conducting CFA using PLS-SEM (SmartPLS). The SmartPLS 3.3.3 software was used to perform CFA, which was used to test discriminant validity. Table 2 shows the CFA results of this study.

**TABLE 2:** Reliability and validity of the construct.

Constructs	Factor loading	Cronbach’s alpha	CR	AVE
**Green alternativeness**		0.948	0.963	0.866
GA1	0.947			
GA2	0.940			
GA3	0.918			
GA4	0.9160			
**Airline corporate image**		0.966	0.972	0.831
GCI1	0.945			
GCI2	0.911			
GCI3	0.913			
GCI4	0.900			
GCI5	0.903			
GCI6	0.898			
GCI7	0.910			
**Green experiential satisfaction**				
GES1	0.935	0.959	0.969	0.860
GES2	0.936			
GES3	0.921			
GES4	0.916			
GES5	0.929			
**Green service fairness**		0.958	0.967	0.855
GSF1	0.952			
GSF2	0.915			
GSF3	0.923			
GSF4	0.915			
GSF5	0.918			
**Airline switching intention**		0.938	0.960	0.889
GSI1	0.957			
GSI2	0.937			
GEI3	0.935			
**Nature experience**		0.936	0.959	0.887
NE1	0.955			
NE2	0.931			
NE3	0.939			
**Self-expressive benefits**		0.937	0.960	0.889
SEB1	0.956			
SEB2	0.940			
SEB3	0.931			
**Warm glow**		0.930	0.955	0.877
WG1	0.949			
WG2	0.936			
WG3	0.925			

*GA, green alternativeness; GCI, airline corporate image; GES, green experiential satisfaction; GSF, green service fairness; GSI, airline switching intention; NE, nature experience; SEB, self-expressive benefits; WG, warm glow; CR, construct reliability; AVE, average variance extracted.*

In this study, Cronbach’s alpha was used as the standard for internal consistency reliability. Cronbach’s alpha was used to calculate the correlation for each path (Choi and Sirakaya, 2006). George and Mallery (2003) indicated that according to the law of alpha reliability, whether the alpha is greater than 0.7 can prove whether the items in the scale are reliable.

According to the operating results of the SmartPLS 3.3.3 software, Cronbach’s alpha values of the eight constructs ranged from 0.930 to 0.966, all exceeding 0.7. The result indicates that the investigated constructs had internal consistency reliability. Composite reliability (CR) is concerned with the internal consistency of the composite factors involving multiple items (Fornell and Larcker, 1981a). CR was used for the reliability test, and the minimum value of CR should be higher than 0.70 (Hair et al., 2010). In this study, the CR values of the eight constructs ranged from 0.955 to 0.972. The reliability test of this study was qualified.

Factor loadings (FL > 0.7) and the average variance extracted (AVE > 0.50) for all items evaluated the effectiveness of the convergence (Fornell and Larcker, 1981a). The FL test was used to determine the measurement validity of the project. Table 2 shows that 33 items of FL ranged from 0.898 to 0.957, which were more than the 0.5 standards. The AVE values of the eight constructs ranged from 0.831 to 0.889, which were greater than 0.50. Moreover, all exceeded the threshold of 0.50 for convergence validity (Fornell and Larcker, 1981a).

In addition, discriminant validity tested the correlation among the square root of AVE (Fornell and Larcker, 1981b). A larger variance of latent variables should be observed in this test. Table 3 presents the inter-construct correlations of the matrix. Correspondingly, all the correlations have satisfied the result. All the correlations imply that the hypothesized measurement model is reliable and valid in structural relations.

**TABLE 3:** Discriminant validity.

	GA	GCI	GES	GSF	GSI	NE	SEB	WG
GA	0.930*							
GCI	0.150	0.912*						
GES	0.211	0.195	0.927*					
GSF	0.194	0.329	0.237	0.925*				
GSI	0.299	0.116	0.246	0.177	0.943*			
NE	0.233	0.153	0.633	0.186	0.238	0.942*		
SEB	0.222	0.226	0.357	0.251	0.134	0.265	0.943*	
WG	0.237	0.569	0.274	0.314	0.133	0.191	0.245	0.937*

*The numbers in the diagonal row are square roots of the AVE.

#### Hypothesis Testing

In this study, SmartPLS 3.3.3 software was used, and PLS-SEM was used to build a structural equation model to test the research hypothesis. The Bootstrapping method was used to ensure the stability of the results.

Table 4 shows the results of the hypothesis test. The results show that warm glow has a significantly positive effect on the airline’s corporate image (β = 0.543, *p* < 0.01). Therefore, Hypothesis 1 is supported. The relationships between self-expressive benefits and green corporate image (β = 0.085, *p* > 0.05) and nature experience and green corporate image (β = 0.026, *p* > 0.05) are not supported in this study. Therefore, Hypotheses 2–3 are not valid. Hypothesis 4 is supported based on the green corporate image that has a significant and positive influence on green experiential satisfaction (β = 0.116, *p* < 0.05).

**TABLE 4:** Direct paths.

	Direct paths	Path coefficient	*t*-value	*P*-value	Hypotheses
H1	Warm Glow - > Airline Corporate Image	0.543	12.457	0.000***	Accepted
H2	Self-Expressive Benefits - > Airline Corporate Image	0.085	1.941	0.052	Rejected
H3	Nature Experience - > Green Corporate Image	0.026	0.627	0.531	Rejected
H4	Airline Corporate Image - > Green Experiential Satisfaction	0.116	2.388	0.017*	Accepted
H5	Green Experiential Satisfaction - > Airline Switching Intention	0.173	3.989	0.000***	Accepted
H6	Green Service Fairness - > Green Experiential Satisfaction	0.168	3.525	0.000***	Accepted
H7	Green Service Fairness - > Airline Switching Intention	0.088	1.908	0.057	Rejected
H8	Green Alternative Attractiveness - > Green Experiential Satisfaction	0.161	3.434	0.001**	Accepted
H9	Green Alternative Attractiveness - > Airline Switching Intention	0.245	5.314	0.000***	Accepted

*p < 0.05, **p < 0.01, ***p < 0.001.

Green experiential satisfaction has a significantly positive effect on green switching intention (β = 0.173, *p* < 0.01). Therefore, Hypothesis 5 is valid. Green service fairness has a significant and positive effect on green experiential satisfaction (β = 0.168, *p* < 0.01) but fails on linking airline switching intention (*p* > 0.05). Therefore, Hypothesis 6 is supported, but Hypothesis 7 is not. The airline alternative attractiveness has a significantly positive effect on green experiential satisfaction (β = 0.161, *p* < 0.01) and green switching intention (β = 0.245, *p* < 0.01). The result verifies Hypotheses 8 and 9.

### Effects of Green Psychological Benefits on Airline Corporate Image

The three psychological benefit determinants, namely warm glow, self-expressive benefit, and nature experience, are widely used to examine the customers’ spiritual comfort of using eco-friendly products or services (Hwang et al., 2019). Starting with these determinants, this study examined their associated effects on the consumers’ viewpoint of green airline image.

Based on the statistics, warm glow is the most significant construct of airline corporate image (Hypothesis 1). This result is also supported by previous studies (Hwang and Choi, 2017). Lin et al. (2017b) also stated that warm glow benefits can affect the green consumers’ perceived value, which has proved to have a strong connection between customers and their green brands. However, as noted earlier, self-expressive benefit and nature experience will not be relearned in the corporate image (Hypotheses 2–3), which does not match the managerial outcome (Hwang and Choi, 2017; Lin et al., 2017a). The results imply that customers might perceive a positive image when they regard themselves as doing the right thing (contributing to the environment). Different from displaying a positive characteristic, a cluster that consumes green aviation is more likely to lay on self-accomplishment (e.g., social responsibility, green brand loyalty, and contribution of pollution abatement). On the contrary, a cluster pays less attention to social belongings or esteem needs when choosing green flight as transportation, such as congruence of self-image appreciation or enjoyment of nature experience. Moreover, our target respondents were Chinese customers who are culturally different from the non-Chinese segment. Their buying decisions are based on low individualism. They are seldom concerned about their self-expressive behavior than the customers who belong to groups that look after each other in exchange for loyalty (Huang and Crotts, 2019).

Nature experience does not support airline corporate image, which agrees with the result of Jamrozy and Lawonk (2017), who indicated that customer purchase intention is up to money worth experience. Today, a highly competitive environment, customer concern price, quality, and value can explain the outcome of the hypotheses that are not supported in relation to nature experience and organization image.

### Effect of Airline Corporate Image on Green Experiential Satisfaction and Switching Behavior

The data analysis reveals that airline corporate image positively influences green experiential satisfaction, and experiential satisfaction further declines airline switching intention (Hypotheses 4–5). This notion concurs with the proposition of Wu and Cheng (2018b). To some extent, experiential satisfaction reflects as a key element for maintaining the existing consumers. Furthermore, the developed corporate image (e.g., professions on developing an environmental reputation and reliable eco-friendly products and services) can reduce the costumers’ switching intention of green brands.

### Relationship Among Green Experiential Satisfaction, Green Service Fairness, Green Alternative Attractiveness, and Airline Switching Intention Perceived by Airline Customers

The third research objective is partially supported. The results indicate that green alternative attractiveness has a significant and positive influence on the airline switching intention (Hypothesis 9). However, green service fairness cannot affect airline switching intention directly (Hypothesis 7). Yieh et al. (2007) highlighted that when a customer perceives the fairness of the price given by the service provider, positive feelings toward the service provider will gradually develop the buying decision. Studies have found that price is a crucial factor for customers satisfaction and loyalty. Therefore, price fairness may determine the customers’ switching intention and come up with loyalty decisions, although they have a relevant green fairness service. The business has become more competitive, customers’ buying decisions are made with corporate brand image, given that the marketer might come up with a green promotion image, which may lead to switching intention. Therefore, a cost-effective flight offering experience satisfaction will ultimately prevail in the rival market.

## Conclusion

This study aims to find the effect of passengers’ perceptions of the green psychological benefits (by examining three dimensions: warm glow, self-expressive benefits, and nature experience), green service fairness, and green alternative attractiveness on its outcome variables in the green Airlines industry. More specifically, this study proposes that the psychological benefit (warm glow, self-expressive benefit, and nature experience) of greenness can affect the airline’s corporate image. In addition, the psychological benefit continues to affect passengers’ green experiential satisfaction, which can eliminate negative intention on airline switching. Meanwhile, this study proposes that green service fairness and green alternative attractiveness have a negative influence on airline switching intention toward green experiential satisfaction. Nine hypotheses were developed from the theoretical relationship among the proposed constructs. The data analysis result includes theoretical and practical implications for stakeholders as follows.

### Theoretical Implications

First, the data analysis indicates that warm glow (Hypothesis 1) is the main driver of the airline’s corporate image. The result supports the previous studies in diverse industries (Hwang and Choi, 2017; Lin et al., 2017b). However, different from previous studies, self-expressive benefit (Hypothesis 2) and nature experience (Hypothesis 3) cannot support the airline corporate image. Although previous studies have confirmed the relationship in a similar field, the result of the current study differs from previous evidence. Utilizing self-expressive benefits and nature experiences for marketing campaigns to improve airline corporate image does not work in China (Lin et al., 2017a,b). As previously mentioned, the Chinese culture reflects low individualism as a seldom concern of self-expressive behavior rather than customer belonging to groups that look after each other in exchange for loyalty. The result implies that not all psychological benefits attract passengers’ notion on airline corporate image. Based on this scenario, the psychological benefit of green carrier choices does not present a self-interested motivation. In contrast, self-achievement when flying with a green airline, can improve the image of an airline organization. One possible explanation is that those who choose green airlines in the market segment in China are those who are concerned about green issues. The main reason for passengers flying *via* a green airline is self-achievement. Another possible reason is that with the development of transportation networks and living standards, people are not required to verbally promise to support environmental protection when they can protect the environment. Thus, the social norm is barely perceptible, and nature experience is not the core benefit for purposeful pursuit.

Second, this study confirms that the airline corporate image supports passengers’ green experiential satisfaction (Hypothesis 4), and green experiential satisfaction can decline the airline switching intention (Hypothesis 5). The importance of green experiential satisfaction and the decline of switching intention have been consistently emphasized in the veracious field. However, applying the existing theoretical concept to a relatively unfamiliar field can improve its reliability and validity (Chen, 2010). Correspondingly, the practical implication in different areas can achieve a more reflective interrelation.

Third, green service fairness (Hypothesis 6) and green alternative attractiveness (Hypothesis 8) have a direct influence on green experiential satisfaction. Green service fairness and green alternative attractiveness can affect airline switching intention through green experiential satisfaction. Green alternative attractiveness can also influence switching intention directly (Hypothesis 9). However, green service fairness fails to affect airline switching intention (Hypothesis 7). To the best of our knowledge, correlations among green experiential satisfaction, green switching intention, green alternative attractiveness, and green service fairness are hardly investigated in the airline industry. The study coincides with the proposition of Wu and Cheng (2018b) who investigated correlations in the green convention. The results may not be accidental. From these relationships, green service can generate the passengers’ satisfaction with a green flight experience. However, green service fairness cannot directly affect the passengers’ switching intention. In this notion, one possible reason is that green service fairness can strengthen the green flight experience. However, choosing whether they will switch to another airline is not the core value for passengers. Wu and Cheng (2018b) stated that those treated unfairly by a green institution may not likely become morally outraged. However, we argue that the participants in our study might not have the associated experience of unfair service treatment. Service fairness, to some extent, is regarded as the service standard for the flight journey. Passengers do not think that service fairness is the reason for them to consider switching. However, service fairness might be one of the core elements for them to evaluate satisfaction of the flight journey.

### Practical Implications

The findings have several managerial implications. First, the psychological benefit of warm glow can decrease consumer switching intention, formulation, and excursion of self-enhancement slogan or other communication strategies. Thus, enhancing the consumers’ warm glow might enhance the corporation image and green experiential satisfaction, thereby, reducing the probability of switching behavior. Hence, brand innovation in the Chinese green airline market should reconsider building their green brand image that can evoke the consumers’ feelings of nature connectedness and moral obligation. On the contrary, marketing should pay less attention to motivating consumers on self-expressive benefits and nature experiences in China and proportionally reduce utilitarian environmental benefits. Lin and Zhou (2020) confirmed the marketing strategy and stated that “utilitarian environmental benefit is evident in the branding of physical goods but fails to support the branding of services.”

Furthermore, green experiential satisfaction can help green service fairness and green alternative attractiveness to contain airline switching intention. Accordingly, green alternative attractiveness can affect airline switching intention directly but not service fairness. Marketers should keep in mind that green service fairness is an essential element for green experiential satisfaction. However, service fairness is not a determinant of consumer switching behavior, because in a competitive environment, pricing is one of the concerns, although the image is green. This notion does not mean that marketers should not pay attention to service fairness. The reason is that once the passengers are unsatisfied because of insufficient demand or unfair service, the disappointment of the flight will conquer satisfaction toward the flight journey, thereby, increasing the consumers’ intention to switch. On the other hand, the competitors of green airlines might provide them with better experiential quality, and they may thus switch to another airline. Notably, in this study, the airline corporations were not limited to the green operation mode. Accordingly, the green airline management should improve the dimensions of experiential quality to allow the consumers to choose green companies and create green loyalty of green airlines.

Lastly, airlines need to improve corporation image and green experiential satisfaction which will result in not only enhancing the brand image but could also reduce the probability of switching behavior as well as establish an eco-surplus culture for the customers.

### Limitation and Future Research

This study has several limitations. First, this study focuses on green marketing constructs, and the relationships are examined in the comprehensive theoretical framework. Other potential green marketing constructs or relationships that are important may have been neglected in the theoretical framework. Future researchers may extend the current theoretical framework and examine whether other potential relationships exist, apart from those identified in this study, in various service industries or other countries.

Second, the data collection was conducted during the COVID-19 pandemic period. The research method was also only limited to a quantitative approach *via* a questionnaire. Moreover, to implement social distancing, we could only reach the participants *via* an online platform. Adopting a mixed-method approach could have been better to minimize the bias of the result.

Third, we only focused on Chinese passengers, which may not be generalizable to other geographical regions in other countries. Hence, future studies should collect samples from different nations to validate the generalizability of our research model. Future studies could also perform a cross-national analysis on the model of this study to determine if the subjects of different nations would generate different results.

## Data Availability Statement

The original contributions presented in the study are included in the article/supplementary material, further inquiries can be directed to the corresponding author/s.

## Author Contributions

SC designed the topic. XZ and YW collected the data and wrote the manuscript. ZL collected the data and reviewed the literature. All authors contributed to the article and approved the submitted version.

## Conflict of Interest

The authors declare that the research was conducted in the absence of any commercial or financial relationships that could be construed as a potential conflict of interest.

## Publisher’s Note

All claims expressed in this article are solely those of the authors and do not necessarily represent those of their affiliated organizations, or those of the publisher, the editors and the reviewers. Any product that may be evaluated in this article, or claim that may be made by its manufacturer, is not guaranteed or endorsed by the publisher.
